# ‘I'm having jelly because you've been bad!’: A grounded theory study of mealtimes with siblings in Australian families

**DOI:** 10.1111/mcn.13484

**Published:** 2023-02-19

**Authors:** Susannah K. Ayre, Melanie J. White, Holly A. Harris, Rebecca A. Byrne

**Affiliations:** ^1^ Woolworths Centre for Childhood Nutrition Research, Faculty of Health Queensland University of Technology South Brisbane Queensland Australia; ^2^ School of Exercise and Nutrition Sciences, Faculty of Health Queensland University of Technology Kelvin Grove Queensland Australia; ^3^ School of Psychology & Counselling, Faculty of Health Queensland University of Technology Kelvin Grove Queensland Australia; ^4^ Department of Psychology, Education and Child Studies Erasmus University Rotterdam Rotterdam The Netherlands

**Keywords:** children, feeding, grounded theory, mealtimes, parenting, qualitative methods, siblings

## Abstract

Obesity prevention interventions have been designed to promote responsive feeding in early childhood. However, existing interventions primarily target first‐time mothers without considering the complexities of feeding multiple children within a family unit. By applying principles of Constructivist Grounded Theory (CGT), this study aimed to explore how mealtimes are enacted in families with more than one child. A mixed‐methods study was conducted with parent–sibling triads (*n* = 18 families) in South East Queensland, Australia. Data included direct mealtime observations, semistructured interviews, field notes, and memos. Data were analysed using open and focused coding, during which constant comparative analysis was applied. The sample comprised of two‐parent families with children ranging in age from 12 to 70 months (median sibling age difference = 24 months). A conceptual model was developed to map sibling‐related processes integral to the enactment of mealtimes in families. Notably, this model captured feeding practices used by siblings, such as pressure to eat and overt restriction, that previously had only been described in parents. It also documented feeding practices used by parents that may occur only in the presence of a sibling, such as leveraging sibling competitiveness and rewarding a child to vicariously condition their sibling's behaviour. The conceptual model demonstrates complexities in feeding that give shape to the overall family food environment. Findings from this study can inform the design of early feeding interventions that support parents to remain responsive, particularly when their perceptions and expectations of siblings differ.

## Key messages

- Through their direct interactions, siblings may impact how they themselves are socialised around food during their early childhood years.
- Siblings may also alter how parents feed their children by virtue of their role within broader family dynamics and structures.
- Obesity prevention interventions should consider the role of siblings in the enactment of mealtimes to support parents to remain responsive, particularly when their perceptions and expectations of siblings differ.

## Graphical abstract

Sibling influences are frequently overlooked at mealtimes, despite recognition of their role in child development and adjustment across a broad range of disciplinary perspectives. By applying principles of Constructivist Grounded Theory (CGT), this study aimed to explore how mealtimes are enacted in families with more than one child. The conceptual model developed in this study demonstrates the complex and integral role of siblings in shaping how parents navigate feeding and lays a foundation for future exploratory and intervention research.

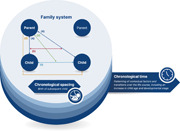

## 1 INTRODUCTION

Infancy, toddlerhood, and early childhood are periods marked by rapid physiological, cognitive, and socioemotional development in which eating behaviours are established (Mura Paroche et al., 2017). While children are born with an innate capacity for appetite self‐regulation, feeding practices used by parents in these early periods may foster or undermine this capacity, and, thereby, affect their long‐term behavioural and weight outcomes (McCrickerd, 2018). However, parent feeding practices are modifiable (Daniels et al., 2015), and, therefore, an appropriate target for early interventions.

In Australia, interventions promoting responsive feeding, such as InFANT in Victoria (Campbell et al., 2008), are now integrated state‐wide into routine preventative health care for parents (Laws et al., 2021). Similar interventions have also been trialled in other states within Australia (Daniels et al., 2009; Wen et al., 2017) and more broadly (Paul et al., 2014; Taylor et al., 2011). However, the evidence supporting these interventions is based primarily on research with first‐time mothers. While a responsive feeding intervention with first‐born infants in the United States (SIBSIGHT) has demonstrated certain spill‐over effects on feeding (Ruggiero et al., 2020), dietary intake (Hohman et al., 2020), and weight status (Savage et al., 2022) of subsequent siblings, it remains unclear as to whether existing interventions are applicable to families with more than one child. To explore this avenue of research, a foundational understanding of how mealtimes are enacted in these families is needed.

Examining feeding relationships with siblings is pertinent, considering that more than half (56.4%–57.3%) of parents in Australia, the United Kingdom, and the United States have two or more children (Australian Bureau of Statistics, 2022; Office for National Statistics, 2022; United States Census Bureau, 2022). As evident in a recent scoping review (Ayre et al., 2022), current research exploring feeding in the sibling context is scarce and reliant mostly on parent‐reported studies. Most of these studies have analysed how differences in parent feeding practices, including restriction, pressure to eat, and monitoring, are correlated with differences in sibling characteristics, including weight status, eating behaviours, temperaments, birth order, and sex. For example, studies have demonstrated that parents tend to more frequently restrict foods from their heavier‐weight child, relative to their lighter‐weight sibling (Berge, Tate, et al., 2016; H. A. Harris et al., 2016; Selzam et al., 2018; Tripicchio et al., 2014). This difference is often attributed to parents' heightened concerns about the dietary intake or weight status of one child (Bosk, 1988).

Evidently, research to date has explored how parents may differentially feed siblings. However, few studies have captured other processes through which siblings may impact the enactment of mealtimes. This gap in the research may stem from a reliance on self‐reported data, which may not capture behaviours that parents are either unaware of, prefer not to report, or are not explicitly asked about in questionnaires or interviews (Pesch & Lumeng, 2017). For example, it has been postulated by Ruggiero et al. (2021) that processes such as resource dilution and learnt experience may alter how parents feed their earlier‐ and later‐born children. These processes are further underpinned by family systems theory (McHale et al., 2012), which is centred on the tenet that the family is a complex, interrelated system that cannot be fully comprehended by analysing subsystems (e.g., parent–child) in isolation from one another (Broderick, 1993). Thus, a better conceptualisation of how parents feed in the sibling context is needed to inform the design of effective family‐based feeding interventions. This is particularly pertinent in early childhood, when parents may have the capacity to mould behaviours, rather than attempt to modify habits that have been embedded over time (St. George et al., 2020). Therefore, the aim of the current mixed‐methods study was to develop a conceptual model that identifies and explores how mealtimes are enacted in families with more than one child between the ages of 1 and 5 years.

## 2 METHODOLOGY AND METHODS

This study was undertaken through an interpretivist lens, informed by social constructionism. This paradigm is underpinned by the notion that knowledge is socially constructed through human interactions (Crotty, 1998). The study further applied principles of Constructivist Grounded Theory (CGT) (Charmaz, 2014), which shares a theoretical framework with Symbolic Interactionism (SI) (Blumer, 1969). While the term ‘constructivist’ was adopted by Charmaz (2014) to acknowledge the subjectivities of researchers and participants in interpreting data, which were reportedly neglected by social constructionist researchers in the late 20th century, there exists a strong alignment between these two theories. SI further asserts that the process of interpretation is mediated by language, gestures, and other symbols, and fundamentally drives human behaviour (Blumer, 1969). Thus, acknowledging the authors' interpretation of the data, CGT was selected as it enabled a rich conceptualisation of the enactment of mealtimes with siblings, for which there is currently inadequate theory. Mealtimes, in themselves, are also replete with symbols of food, rituals, conversations, and seating arrangements, thus providing a window into patterns of significant social interactions (Fiese et al., 2006).

### 2.1 Positionality

Recruitment, data collection, and data analysis were undertaken primarily by the first author (SA). This author is a female dietitian and PhD candidate who has undertaken postgraduate coursework in qualitative research. Before the commencement of the study, the authors had no academic relationship with the participants, and the overall aims of the research were disclosed to them. It is acknowledged that the positioning of the author as a dietitian may have impacted discussions with participants. However, the author used reflexive practice to ensure that this did not override participants' own perceptions of mealtimes, and offered reassurance of the nonjudgemental nature of both the observations and interviews.

### 2.2 Design

The study used a qualitative mixed‐methods design. While both parents provided consent on behalf of themselves and their children to participate, data collection and analysis focused primarily on one parent and two children, with mothers being the nominated parent in almost all (94.4%) families. Informed consent was also provided by all other adults captured in the mealtime observations. In three (16.7%) families, both parents were included in the analysis of mealtime observations due to their relative contributions to feeding. Similarly, in two (11.1%) families, both parents opted to participate in the interview. Families were compensated with an AU$50.00 e‐gift card for their participation. The Consolidated Criteria for Reporting Qualitative Research (COREQ) Checklist was used for reporting (Tong et al., 2007) (Supporting Information: Table S1). To maintain participant confidentiality, codes and pseudonyms were used when reporting direct quotations.

### 2.3 Participants and recruitment

The study was conducted with 18 parent–sibling triads (*n* = 54 participants) in South East Queensland, Australia. Eligible parents were aged 18 years or older, used English as their primary language, and had two or more children aged 1 to 5 years. Parents were excluded if at least one of their participating children did not reside with them full‐time or were diagnosed with a congenital abnormality or chronic condition that affected their dietary intake and growth. If parents had more than two children within this age range, the two eldest children were considered the participants.

Purposive, snowball, and theoretical sampling were used for recruitment. Digital advertisements were distributed through childcare centres using their online communication software and local parent groups on Facebook. Parents registered their interest online and were subsequently contacted via telephone or email. Following their participation, parents were asked to distribute the advertisement to any contacts who fit the eligibility criteria.

As data collection and analysis advanced, theoretical sampling was also undertaken using the recruitment methods outlined above. In line with methods explicated by Butler et al. (2018), theoretical sampling necessitated the use of two strategies: sampling for specific participant attributes and the inclusion of additional interview questions (Supporting Information: Table S2). To explore concepts emerging from the data in relation to the impacts of age and age gaps on sibling feeding dynamics, the eligibility criteria were amended to recruit parents of twins (*n* = 2 families) and siblings aged 3 to 5 years (*n* = 3 families).

### 2.4 Data collection

**Figure 1:**
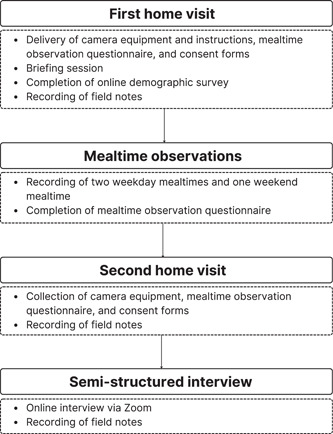
Flowchart of participant involvement in data collection.

Data collection was undertaken between August 2021 and April 2022. Data in this study comprised naturalistic mealtime observations, semistructured interviews, field notes, and memos. CGT is compatible with multiple methods of data collection and lends itself to those that capture the attitudes, intentions, and actions of participants, and the contexts in which they are enacted (Charmaz, 2014). The data collection period for individual families varied (median: 3.0 weeks, IQR: 2.0–4.1 weeks), and was staged to account for the availability of equipment and allow for concurrent data analysis to occur, which is central to CGT (Charmaz, 2014). For each family, two home visits were scheduled to deliver and collect all necessary equipment and forms (Figure 1). Parents video‐recorded three mealtimes over approximately 2 weeks (median: 1.8 weeks; IQR: 1.0–2.8 weeks) and participated in an online interview approximately 1 week later (median: 1.0 weeks; IQR: 0.4–1.3 weeks). Field notes were taken at each point of contact to record observations. Data collection continued until all authors determined that the concepts were adequately explored and substantiated, as new data revealed no additional properties (Charmaz, 2014).

#### 2.4.1 Sociodemographic data

During the first home visit, parents completed an online demographic survey via REDCap (Research Electronic Data Capture) (P. A. Harris et al., 2009, 2019), collecting household composition and postcode; parental age, gender, culture and ethnicity, and educational attainment; in addition to sibling birth date and sex.

#### 2.4.2 Observations

A digital video camera and tripod were provided to each family. During the first home visit, nominated parents were briefed on the mealtime observation protocol. It was requested that parents record three evening mealtimes, two on weekdays and one on a weekend, as they typically occurred in their homes. Parents were asked to record the first video on a weekday. Parents were also provided with a hard‐copy questionnaire to document the date and time of the recorded mealtimes, and briefly describe who was present and what was served. They were also asked to score how typical each mealtime was on a scale from 1 (‘very atypical’) to 10 (‘very typical’) and outline contextual factors that contributed to lower scores. Upon completion of the mealtime observations, the camera and forms were collected and reviewed.

#### 2.4.3 Interviews

Video‐recorded semistructured interviews were conducted with parents online via Zoom videoconferencing software (Zoom Video Communications, 2022). The interview protocol was developed by SA and refined in collaboration with RB (PhD), HAH (PhD), and MW (PhD) (Supporting Information: Table S2).

### 2.5 Data analysis

Demographic data were analysed using descriptive statistics in SPSS (Statistical Package for the Social Sciences) Version 27 (IBM Corp, 2020). Postcodes were used to determine Index of Relative Socioeconomic Advantage and Disadvantage (IRSAD) scores (Australian Bureau of Statistics, 2018) and the proportion of children considered developmentally vulnerable in two of the five Australian Early Development Census (AEDC) domains (Australian Government, 2018) for each respective suburb. Sibling birth dates were used to calculate ages and relative age gaps.

**Figure 2:**
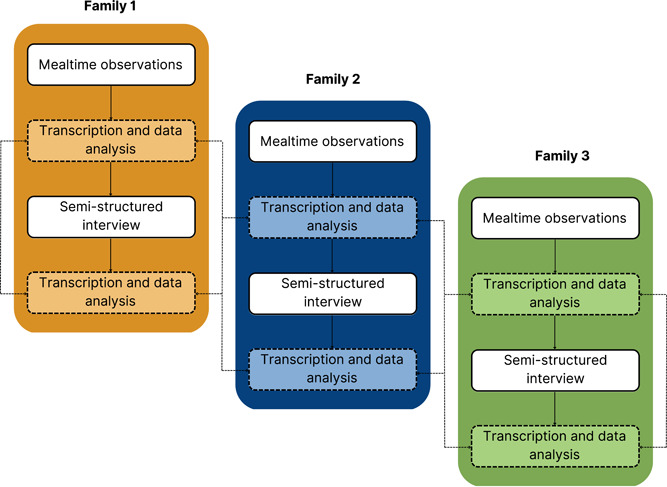
Example flowchart of concurrent data collection and analysis procedures, with constant comparative methods applied within and between families.

Data collection and analysis were conducted iteratively. The second and third mealtime observations were subject to analysis, while the first observation served as an opportunity for participants to become habituated to the camera. Parents were unaware of this fact. Due to deviations from the protocol, exceptions were made for two (11.1%) families to ensure that variation over weekday and weekend mealtimes was captured. Mealtime observations and interviews were transcribed verbatim and analysed in NVivo Version 1.5.2 (QSR International, 2020). Transcripts were analysed using initial and focused coding. Initial coding involved coding the data line‐by‐line with gerunds, which are verbal nouns that capture participant actions (Charmaz, 2014). In focused coding, relevant codes were selected to form more abstract categories to synthesise the data. Using a purposeful approach (Boeije, 2002), the constant comparative method was used to compare data at each stage of coding (i.e., transcripts, codes, categories). For example, a code constructed from one interview excerpt was compared with codes constructed from different excerpts within the same interview, as well as excerpts from associated mealtime observations. As additional participants were recruited, comparisons were also drawn between existing and incoming data. Figure 2 includes a flowchart demonstrating this procedure, which was applied across the whole sample. Memos were written during this process to refine emerging codes and categories. As the analysis proceeded, a preliminary model was constructed and compared to relevant theory in the literature, heightening the authors' theoretical sensitivity (Charmaz, 2014).

To enhance rigour, reflexive journal notes and memos were written by SA to document assumptions and biases, and maintain an audit trail of coding decisions. These decisions were also discussed on a frequent basis among all authors. In addition, approximately 10% of the interviews and mealtime observations were coded independently by RB and/or MW for the purpose of incorporating alternative perspectives, rather than achieving consensus.

### 2.6 Ethical statement

Ethics approval for this study was obtained by the Queensland University of Technology Human Research Ethics Committee on 05 August 2021 (reference number: 4049). All parents provided informed consent on behalf of themselves and their children to participate. Informed consent was also provided by all other residents captured in the mealtime observations.

## 3 RESULTS

### 3.1 Sample

Overall, the online screening and registration form was completed by 57 parents, of whom 18 (31.6%) agreed to participate. Data on reasons for nonparticipation were not collected. However, anecdotally, reasons cited by parents included time constraints and reluctance to be video recorded. The final sample comprised mostly white two‐parent families, each with at least two children. The sociodemographic characteristics of the sample are summarised in Table 1.

**Table 1:** Sociodemographic characteristics of the sample (*n* = 18 families).

Household‐level data	*N* (%)
Household composition	
Number of adults (≥18 years)	
Two	16 (88.9)
Three^a^	2 (11.1)
Number of children (≤12 years)	
Two	10 (55.6)
Three	6 (33.3)
Four	2 (11.1)
AEDC developmental vulnerability score based on postcode^b^	
Below national average	15 (83.3)
Above national average	3 (16.7)
IRSAD score based on postcode^c^	
Quintile 5	10 (55.6)
Quintile 4	4 (22.2)
Quintile 3	2 (11.1)
Quintile 2	2 (11.1)
Quintile 1	0 (0.0)

Parent‐level data	Mothers (*n = *18)	Fathers (*n *= 18)
	Mean (SD)	
Age (years)	36 (2)	38 (5)

	*N* (%)	
Cultural/ethnicity group		
Australian	14 (77.8)	13 (72.2)
Asian	2 (11.1)	1 (5.6)
European	0 (0.0)	2 (11.1)
North American	1 (5.6)	0 (0.0)
Mixed	1 (5.6)	2 (11.1)
Highest level of education		
University degree	17 (94.4)	13 (72.2)
Certificate or diploma	1 (5.6)	3 (16.7)
Year 10 or below	0 (0.0)	2 (11.1)

Child‐level data	All siblings (*n *= 36)	Earlier‐born siblings (*n *= 18)	Later‐born siblings (*n *= 18)
		Median (IQR)	
Age (months)	41.9 (31.1–58.8)	57.2 (42.9–61.9)	31.1 (22.7–38.4)
Age gap between siblings (months)	23.5 (19.0–30.1)	‐	‐

	***N* (%)**		
Births			
Single births	16 (88.9)	‐	‐
Multiple births (i.e., twins)	2 (11.1)	‐	‐
Sex			
Girl	13 (36.1)	8 (44.4)	5 (27.8)
Boy	23 (63.9)	10 (55.6)	13 (72.2)
Sibling composition by sex^d^			
Boy–boy	7 (38.9)	‐	‐
Girl–girl	2 (11.1)	‐	‐
Boy–girl	3 (16.7)	‐	‐
Girl–boy	6 (33.3)	‐	‐

Abbreviations: AEDC, Australian Early Development Census; IQR, interquartile range; IRSAD, Index of Relative Socioeconomic dvantage and disadvantage; SD, standard deviation.

^a^ Included an au pair or child aged ≥18 years.

^b^ Determined based on the proportion of children in each suburb considered developmentally vulnerable on two of the five ADEC domains.

^c^ Calculated based on 2016 Statistical Area Level 1 (SAL1) data with Quintile 1 indicating the ‘most disadvantaged’ areas and Quintile 5 indicating the ‘most advantaged’ areas.

^d^ Listed in order of earlier‐born to later‐born sibling.

### 3.2 Feeding dynamics

Mealtime and interview characteristics are summarised in Table 2. Data supporting five constructs are reported here. Each construct describes a process through which siblings may either *directly* or *indirectly* (i.e., via their parents) influence the socialisation of children around food. Following this, a conceptual model is presented to explain how these constructs may interact to influence the enactment of mealtimes in families with more than one child.

**Table 2:** Characteristics of mealtime observation and interview data.

Mealtimes observations (*n* = 36)	*N* (%)
Persons present	
Mothers	36 (100.0)
Fathers	35 (97.2)
Other siblings	14 (38.9)
Relatives outside of the immediate family^a^	1 (2.8)

	Median (IQR), range
Typicality scores^b^	8 (8–9), 3–10
Duration (min)	25.99 (20.95–35.31), 13.07–61.20

Interviews (*n* = 18)	***N* (%)**
Persons present	
Mother	16 (88.9)
Mother and father	2 (11.1)

	**Median (IQR), range**
Duration (min)	51.40 (43.12–59.19), 33.52–91.80

Abbreviation: IQR, interquartile range.

^a^ Included a grandmother.

^b^ *n* = 1 missing datum.

#### 3.2.1 Direct sibling influences

(1) *Siblings interacting*

The nature of sibling interactions at mealtimes varied. First, the siblings served as companions for one another by engaging in storytelling and play. These interactions were often centred around food. For example, during one mealtime, an older sister (4‐year‐old) engaged her younger brother (2‐year‐old) in pretend play by using her ‘magic asparagus’ as a wand to transform him into different animals (Family 12). Siblings also collaborated and conspired with one another during mealtimes. For example, parents described how siblings traded foods for mutual reward or to ‘clean their plates’. One mother with a 5‐year‐old boy and a 3‐year‐old girl explained:

Interview excerpt (Family 18):

Mother: They'll often trade [food with] each other in order to get their plate empty…so that they can go off and play or watch a movie.

Second, parents perceived that their children learned to eat through observation of their siblings. This was usually described in relation to the younger sibling, for whom parents attributed an increased willingness to accept a wider variety of foods to being exposed to an older sibling who modelled this behaviour. However, the tendency of children to imitate behaviours also had implications for the enactment of mealtimes. For example, children tended to mimic more disruptive behaviours, such as playing with food or cutlery, and leaving the dining table before they had finished eating.

Siblings also assumed a parenting role, through which they facilitated eating at mealtimes. This was particularly evident in older siblings, who tended to enact the feeding practices or intentions that were endorsed by their parents. For example, siblings were observed to educate one another about the sensory or nutritional properties of a food. During one mealtime, an older brother (4‐year‐old) provided reassurance to his younger brother (2‐year‐old) that the seeds in his kiwi fruit were safe to eat:

Mealtime excerpt (Family 9):

Isaac (2‐year‐old): Why's it got black in it? Right in the middle! Why has‐? [points to centre of kiwi fruit].

Jake (4‐year‐old): Isaac, black, black is seeds and you can eat the seeds.

Isaac (2‐year‐old): Black is the seeds… and we can eat kiwi fruit seeds, but not ma‐, mango seeds!

Jake (4‐year‐old): No [shakes head].

In a separate mealtime, an older sister (5‐year‐old) negotiated and reasoned with her younger sister (3‐year‐old) to persuade her to eat:

Mealtime excerpt (Family 14):

Isabel (5‐year‐old): Just one? Try! [offers slice of pork to Mia].

Mia (3‐year‐old): [Places pork into mouth].

Isabel (5‐year‐old): Is it nice?

Mia (3‐year‐old): Yuck [shakes head; spits out pork].

…

Isabel (5‐year‐old): How about this one? [offers slice of pork to Mia].

Mother: C'mon Mia. It's good for your muscles.

Mia (3‐year‐old): [Shakes head].

Isabel (5‐year‐old): I'm eating mine. It's good for your muscles. Just have it. How about this Mia? [halves slice of pork]. Here. You have this bit. I'll have this bit [places slice of pork into Mia's bowl].

The siblings also offered one another prompts and advice on how to behave at mealtimes. For example, older siblings were observed teaching their younger siblings how to use cutlery and correcting their manners.

Furthermore, siblings impacted the preferences and behaviours of one another by serving as sources of comparison and competition. For example, children explicitly identified factors that placed them at a perceived relative disadvantage to their sibling. These factors included the portion size and type of foods served, and the level of attention received by their parents. This process of comparison was evident when an older sister (5‐year‐old) questioned why she was served a smaller portion of noodles compared to her younger sister (3‐year‐old):

Mealtime excerpt (Family 14):

Isabel (5‐year‐old): Why does Mia get more than me?

Mother: I don't know that she has had her food. I think she's just a bit too tired to eat.

Isabel (5‐year‐old): No, she got more!

The implications of these comparisons on food acceptability were evident when children perceived themselves to be at a disadvantage. A mother with two boys (5‐year‐old and 2‐year‐old) explained:

Interview excerpt (Family 10):

Mother: You'll make something, and they just refuse to eat it, or they start having tantrums and crying because, ‘Henry got the slightly bigger banana’… or ‘He got four grapes, and I got three grapes.’

These implications were also evident during a mealtime when children were served different foods to one another:

Mealtime excerpt (Family 15):

Mother: Do you want [an enchilada], like what we had last night?

Evie (5‐year‐old): [Nods].

Mother: …Here, Evie. C'mon darling, come sit here. In you get [serves enchilada to Evie].

Max (3‐year‐old): I want some of that!

Mother: No, this is what you're having, okay? [gestures towards noodles in Max's bowl].

Max (3‐year‐old): But I want some of that [speaks with whiny tone].

In contrast, siblings also differentiated themselves from each other based on these comparisons. For example, one mother explained that her older child (5‐year‐old boy) tended to refuse foods that her younger child (2‐year‐old boy) had expressed a liking for:

Interview excerpt (Family 10):

Mother: George won't eat blueberries because Henry likes them, and he won't have anything with honey on it because Henry likes honey… You buy apples, but Henry wants an apple, so George is like, ‘Why didn't we get pears? I won't eat that’.

In some families, children were contrasted against one another in terms of their behaviours, with one child assuming an ‘idealistic’ role or status within the family. During mealtimes, siblings were observed to reinforce these identities by, for example, drawing attention to their own behaviours when their parents had corrected that of their sibling:

Mealtime excerpt (Family 1):

Lily (3‐year‐old): I don't like it [speaks with whiny tone].

Mother: Open. Don't care. Open [holds fork towards Lily's mouth].

Lily (3‐year‐old): [Whines; leans backwards].

Hannah (5‐year‐old): …I don't like it, and I'm eating it [speaks with boastful tone].

Mealtime excerpt (Family 16):

Elijah (3‐year‐old): [Screams; picks up fork and throws it across the table].

Mother: I don't know if Elijah's getting any jelly after that.

…

Lucas (3‐year‐old): Mum, can I have some jelly? Can I have some jelly?

Mother: Yes mate, you can have some jelly [serves bowl of jelly to Lucas].

Lucas (3‐year‐old): Red jelly! [speaks with excited tone].

Elijah (3‐year‐old): …I want jelly too! [speaks with angry tone].

…

Lucas (3‐year‐old): I'm having jelly because you've been bad!

Finally, the competitive nature of the sibling relationship was evident during mealtimes. Siblings were observed to compete for both tangible (e.g., food, cutlery, chairs) and nontangible (e.g., parental attention) resources. This was evident during a mealtime when two brothers (4‐year‐old and 2‐year‐old) served themselves from a bowl positioned in the centre of the dining table:

Mealtime excerpt (Family 9):

Jake (4‐year‐old): [Serves chicken onto own plate].

Isaac (2‐year‐old): Don't have all of them! Don't have all, all of them on your plate. Don't have all of them! My bit! [yells].

Jake (4‐year‐old): Well, you can't have all of them.

Mother: There's plenty more if we eat all of that, okay?

Jake (4‐year‐old): If you eat all of that, there's more. But you cannot have that much [slides bowl of chicken towards Isaac].

Furthermore, children were aware of receiving more favourable treatment from their parents when compared to their siblings. This was evident, for example, when an older brother (3‐year‐old) was permitted certain foods that his younger sister (1‐year‐old) was not:

Interview excerpt (Family 2):

Mother: Cooper gets Milo® for breakfast. For Emma, we try and hold off as much as possible. Emma won't get Milo® until maybe she's about 3 years old… He knows it's something special for him… He will say, ‘This is mine, it's not for Emma’.

#### 3.2.2 Indirect sibling influences

(2) *Parents using siblings as mediators of feeding practices*
  During mealtimes, parents played on sibling dynamics to influence the amount or types of food that they consumed. For example, parents capitalised on opportunities for observational learning between siblings. This was achieved by explicitly drawing attention to the sibling who acted as a role model for the target child (i.e., the child whose behaviours parents intended to modify). For example, one father reinforced the behaviours of his older child (4‐year‐old boy) to demonstrate to his younger child (2‐year‐old boy) how he should eat his food:
  Mealtime excerpt (Family 11):
  Father: Look what Samuel's doing. He's gobbling up his pumpkin and his mango because he knows that he can like it. You like your pumpkin. Sit down. Have a go.
  However, on occasions, parents also positioned the target child as the role model for their sibling, with the intention of motivating them to continue a behaviour. For example, one mother explained to her older child (3‐year‐old girl) that if she continued eating her berries, then her younger brother (1‐year‐old) might also eat his:
  Mealtime excerpt (Family 8):
  Julia (3‐year‐old): I like blueberries.
  Mother: I know. Yours has got blueberries and blackberries in it. How's Riley going with the berries? [looks at Riley]. Is it good?
  Riley (1‐year‐old): [Winces].
  Mother: No. I bet you spit them out [smiles].
  Riley (1‐year‐old): [Spits blueberries out onto plate].
  Mother: …Yeah [smiles; looks at Julia]. He'll watch you though. If you eat, he might eat. He might do it too.
  In line with this notion, parents often reported intentions of targeting the behaviour of one child, typically the older sibling, with the expectation that their younger sibling would then imitate them. One mother of three children (including a 4‐year‐old boy and 2‐year‐old boy) explained that by persuading one child to eat, generally, the others would then ‘follow suit’ (Family 7). On the contrary, if a child exhibited overt and emotional food refusals during a mealtime, parents often attempted to separate or divert attention from them to avoid spill‐over effects onto other siblings.
  Parents also engaged in vicarious operant conditioning, a practice in which opportunities are constructed for children to observe others acquiring a response in exchange for behaviour to learn that they, too, can obtain that same response. This was achieved by overtly rewarding the behaviour of a sibling with the intent of modifying the subsequent behaviour of the target child. As evident in the following mealtime excerpts, this practice may involve the use of tangible rewards, such as food:
  Mealtime excerpt (Family 1):
  Lily (3‐year‐old): I don't like it [speaks with whiny tone].
  Mother: If you're not eating it, then you're just going to get down.
  Hannah (5‐year‐old): Yeah. It's really yum.
  Mother: Finished? Get down then…
  Lily (3‐year‐old): [Climbs down from chair].
  …
  Mother: Hannah's getting an ice block… Just wash your hands and then you're going up to play while Hannah finishes her dinner and gets an ice block.
  Mealtime excerpt (Family 17):
  Leo (5‐year‐old): Can I have cheese please?
  Mother: Can you push your bowl this way please? [sprinkles cheese onto Leo's pasta].
  James (5‐year‐old): I want some more cheese.
  Mother: When you eat some more dinner. He's finished, see [points to Leo's bowl].
  In contrast, intangible rewards may also be used. For example, one mother of twins (3‐year‐old boys) explained how she redirects attention and praise onto the child who is eating during a mealtime when their sibling misbehaves:
  Interview excerpt (Family 16):
  Mother: I'll just say, ‘Oh, Elijah, that's really nice, isn't it? You like it…’ It's ignoring the bad behaviour and asking the other one to tell us something… [or] praising the other one for eating, you know? Just giving them attention.
  In the sample, siblings also occasionally served as active mediators of their parents' feeding practices. One mother, for example, explained how she prompts her older child (5‐year‐old girl) to persuade her younger child (3‐year‐old girl) to eat:
  Interview excerpt (Family 1):
  Mother: Sometimes I will also give Hannah a bit of a heads up actually. I'm like, ‘Look, you're going to love this meal. I know you're going to love it. You've got to tell Lily that you're going to love it, okay? And that it's really yummy, okay?’ And she does, she's like, ‘Mm, Mum, I'm loving this’.
  Similarly, one mother was observed encouraging her older child (3‐year‐old girl) to praise her younger child (1‐year‐old boy) for tasting his food:
  Mealtime excerpt (Family 8):
  Riley (1‐year‐old): [Bites mango].
  Mother: Good boy. Say, ‘Good trying Riley!’
  Julia (3‐year‐old): Good trying Riley.
  On occasions, this practice was applied in a more implicit manner, with parents providing a motive for the sibling to, of their own accord, enact these practices. For example, one mother of four (including a 5‐year‐old boy and 3‐year‐old) explained that she stipulates to her children that dessert will not be served until they have all finished eating their dinner, which often prompts them to motivate one another to eat more quickly:
  Interview excerpt (Family 18):
  Mother: We say to them, ‘Everyone's got to finish the meal before we have cake.’ And they're really good at encouraging each other. [They'll say to each other], ‘Come on, you've got to finish that so we can have cake’.
  This practice was also observed during a mealtime, resulting in an older brother (5‐year‐old) attempting to persuade his younger brother (2‐year‐old) to finish his dinner, so that they could both be served dessert:
  Mealtime excerpt (Family 10):
  George (5‐year‐old): Can I please have a little bit of cake?
  Mother: Not until Henry's finished his food.
  …
  George (5‐year‐old): Henry, do you want cake or not?
  Henry (2‐year‐old): [Shakes head].
  …
  George (5‐year‐old): Do you know what it has in it? Carrot! And you've eaten like all of your carrot. Nearly. And if you want cake, you have to eat up.
  While infrequent, parents also leveraged the competitive nature of the siblings to motivate them to eat. This involved, for example, challenging the siblings to race one another in eating at mealtimes. One mother with two boys (4‐year‐old and 2‐year‐old) explained how this practice was particularly effective in motivating her older child to eat:
  Interview excerpt (Family 9):
  Mother: Jake is very motivated by competition. Isaac doesn't really understand it yet… I guess in using the competition tactic, we're actually trying to get Jake to do something so that Isaac will copy him… [I have said to them before], ‘See who can eat the sausage first…’ and ‘Who can have the first mouthful?’
  This practice was also observed during a mealtime when a mother with twins (5‐year‐old boys) identified when one sibling had almost finished their food, in an attempt to urge the target child to eat more quickly:
  Mealtime excerpt (Family 17):
  James (5‐year‐old): [Cries; puts head in hands].
  Mother: …Leo has nearly finished, James. You need to eat your food.
  Similarly, parents tended to offer leftover food from one child to another, occasionally with the intention of motivating that child to finish the food themselves due to their innate reluctance to share with their sibling. For example, one mother described how her older child (3‐year‐old boy) tended to eat more when she threatened to serve his leftover food to his younger sister (1‐year‐old):
  Interview excerpt (Family 2):
  Mother: I think also it motivates him to eat as well. What we do sometimes, and I don't recommend this, is [we say to him], ‘Well, if you don't want it, then Emma can have it…’ He doesn't want his sister to have it, so he eats it.
  This practice was also observed during a mealtime when a younger brother (2‐year‐old) ate food that he initially refused to avoid it being served to his older brother (4‐year‐old):
  Mealtime excerpt (Family 7):
  Mother: Okay, are you eating anymore?
  Liam (2‐year‐old): [Whines; shakes head].
  Mother: Okay, well Oliver's going to eat it [picks up slice of avocado from Liam's plate and offers it to Oliver].
  Liam (2‐year‐old): No! [snatches avocado and places it on own plate].
  Mother: Well then, eat it.
  Liam (2‐year‐old): No.
  Mother: You don't get to not eat it when your brothers will happily eat it [picks up slice of avocado from Liam's plate].
  Liam (2‐year‐old): No! No, don't! No, don't! No, don't! [yells].
  Mother: Well then, eat it [offers avocado to Oliver].
  Liam (2‐year‐old): [Snatches avocado and holds it in hand].
  Mother: [Picks up different slice of avocado from Liam's plate and offers it to Oliver].
  Liam (2‐year‐old): No! No, don't! No! No, don't!
  …
  Mother: Okay, put it in your mouth [offers avocado to Liam].
  Liam (2‐year‐old): Don't give it to Oliver [speaks with angry tone; takes avocado from fork and eats it].
  Finally, parents attempted to entice the target child to eat by using language that depicted their food as superior to that of their siblings. This practice was observed during a mealtime when a mother with a 3‐year‐old girl and 1‐year‐old boy used comparative language to describe the food that she was serving to her older child (e.g., ‘*I got you the longest chip!’*) (Family 4).
(3) *Parents moderating sibling dynamics through feeding practices*
  In contrast, parents attempted to moderate sibling dynamics by minimising competition and conflict between them. For example, parents had intentions of distributing tangible (e.g., foods) and nontangible (e.g., attention) resources fairly among siblings. These intentions may serve to reduce the strength of the reaction of children in response to other feeding practices in which they are treated differently. In addition, they may increase the practicality of mealtimes, with parents often describing their reluctance to serve different foods to each child due to the additional cognitive and physical workload required to execute this. However, as demonstrated by a mother of two girls (5‐year‐old and 3‐year‐old), food selection may be limited by the preferences of a fussier child, which may then have implications for their sibling, such as decreased exposure to novel foods:
  Interview excerpt (Family 1):
  Mother: Knowing that Hannah is easier to persuade and usually eats most foods I give her, I don't really tailor to her too much. Unfortunately, she gets what she's given. I do tailor it more towards what I know Lily will eat just to keep mealtimes a bit calmer… But I try to make it look like I'm having the same approach with both, like I will give them the same food and they will eat at the same time.
  Intentions of distributing resources fairly tended to vary by sibling age. For example, younger siblings transitioning into toddlerhood often received more attention due to their relative degree of dependence. However, as the gap between siblings' cognitive and physical skills narrowed, parents were more inclined to assimilate them in the context of feeding. These intentions also prompted some parents to question whether they *should* adopt certain feeding practices (e.g., restriction) for their children due to differences in their age or stature. One mother with girls (5‐year‐old and 3‐year‐old) explained:
  Interview excerpt (Family 14):
  Mother: They basically eat the same amount of food. I'm always conscious, do I give them the same amount? They're almost the same size, but Mia's like half her [Isabel's] age. It's quite difficult because Mia also thinks she has been short‐changed if she sees that Isabel has more, even though she's smaller or should be smaller.
  Furthermore, some parents felt compelled to rectify behaviours in their children that generated competition between them. One mother with a 5‐year‐old boy and 3‐year‐old girl explained:
  Interview excerpt (Family 18):
  Mother: We definitely had to work on getting rid of the competitiveness of the eating situation and teaching them just to enjoy their food. You know, ‘It's not a race. Let's just enjoy it’.
  These dynamics also varied depending on the age differences between the siblings. For example, one mother with twins (5‐year‐old boys) discussed how she avoided comparing her children because there was no age difference between them:
  Interview excerpt (Family 17):
  Mother: If you've got a 4‐year‐old and a 3‐year‐old and you're saying to the 3‐year‐old, ‘Look at how well the 4‐year‐old is eating,’ …it's different because they're a little bit older. The 3‐year‐old knows they've got that growing time. Whereas with the boys, because they're the same age, I kind of feel like if I'm doing that, then I'm saying, ‘Well, he's being the good child and you're being the bad child because you're both the same age’.
  Therefore, attempts to moderate sibling dynamics may be particularly apparent in parents with shorter spacing between child births, and whose children are older, and, therefore, more comparable in terms of their developmental capabilities.
(4) *Parents adapting feeding practices in response to siblings*
  Differences in parent feeding practices for siblings, albeit often subtle, were also evident in the sample (Supporting Information: Table S3). These differences were contingent upon parents' perceptions of each child in relation to their developmental stage, eating behaviours, food preferences, temperaments, and other contextual factors. For example, parents described pressuring their older child to eat more, providing more leniency with the food options provided to their fussier child, and enforcing a less structured mealtime setting for their child who was more easily distracted by stimuli. Thus, differences were observed across a broad scope of practices, including those which may impact the overall functionality of the mealtime. This was epitomised by one mother with a 5‐year‐old girl and 2‐year‐old boy, as she discussed her feeding priorities:
  Interview excerpt (Family 3):
  Mother: Priority number one is to feed Harper. Two, is trying to get food into Miles over a bottle. And then three, we eat.
  While parents were asked to compare the characteristics of their children in the interviews, it was evident that these comparisons also impacted the value judgements that parents placed upon them. For example, one mother explained how the eating behaviours of her younger child (2‐year‐old boy) altered the perceptions that she had of her older child (4‐year‐old girl):
  Interview excerpt (Family 12):
  Mother: She's always been a pretty good eater, but I guess I thought she could always do with improvement until we had Jeremy. He's a bit more picky, so Charlotte looks like quite a good eater beside Jeremy some days.
  In addition, while parents described differences in how their children responded to feeding practices, these differences were often attributed to dissimilarities in early feeding methods, or characteristics beyond parental control, such as the age, sex, and genetic makeup of the siblings.
(5) *Parents' evolving feeding practices with the inherent restructuring of sibship size and positioning*

Parents further described how their feeding practices had evolved with the inherent restructuring of sibship size and positioning that occurred following the birth of their younger child. Two processes were evident: diluting resources and learning through experience.

First, in this sample, parents acknowledged the finite nature of time and economic resources. Thus, as families expanded, the resources that were available to any one child declined. For example, parents were required to divide their attention among the siblings, which resulted in fewer parent‐child feeding interactions. One mother with a 3‐year‐old girl and 1‐year‐old boy discussed:

Interview excerpt (Family 4):

Mother: With Lucy, there could be so much more like, ‘Look at the colour of the peas…’ You could narrate a little bit more and teach her about things… Obviously when you've got two of them to try and manage, that level of engagement individually is a little bit different.

It was evident in the sample, however, that to overcome the impacts of resource dilution, parents needed to adapt their feeding practices. This involved renegotiating and reallocating resources to balance the needs of all family members. For example, one mother with four children (including a 5‐year‐old boy and 3‐year‐old girl) explained how she distributed responsibilities among the siblings at mealtimes:

Interview excerpt (Family 18):

Mother: With the bigger family, I think there are definitely some things that our kids take on that I know that their friends definitely don't do. Like the kids, they help unload the dishwasher, they take out the recycling, all things to do with obviously preparing meals.

On the contrary, parents were often less inclined to involve multiple children in more time‐ and resource‐intensive activities, such as grocery shopping and food preparation. This was often due to the difficulty in coordinating and monitoring these activities with siblings, particularly when they differed in terms of temperament or developmental capability. According to one mother with three children (including 5‐year‐old twin boys), this often resulted in ‘too many cooks in the kitchen’:

Interview excerpt (Family 17):

Mother: When you've got three helping out, you've got to know what each of them is doing. You've got to be thinking about what they're going to do next so that when they're finished, you can give them another task, as well as watching the other two, as well as watching what you're doing… There's just a lot of thought that would need to go into that.

As families expanded, parents also tended to implement a more structured mealtime routine, as well as prioritise time‐ and cost‐efficient meals. For example, one mother with a 5‐year‐old boy and 3‐year‐old girl explained how meal selection in her family had evolved over time, with an increased focus on convenient meals prepared in ‘bulk’:

Interview excerpt (Family 18):

Mother: Our meals have become, I'm going to say, a lot more basic. I used to do chicken Pad Thai and lots of prep work for meals. We used to make our own pizza dough and things like that… We've definitely had to go, ‘Okay, well, let's make life a little easier, and do something that can be made in bulk with not as many preparation steps…’ Yeah, so we definitely simplified the menu a lot more.

Second, differences in parent feeding practices with younger and older siblings also resembled a shift in expectations and priorities. With the novelty of the firstborn child and transition into parenthood, parents often described their approach as more cautious, vigilant, and strict. However, through this experience, parents found reassurance in their child's capacity for appetite self‐regulation, which enabled them to engage in more responsive feeding practices with subsequent siblings. For example, one mother explained that through her experience with her older child (4‐year‐old boy), she learnt to become more accepting of food refusal as a normal developmental phase in children, which then impacted her attitude towards feeding her younger child (2‐year‐old boy):

Interview excerpt (Family 7):

Mother: I was a really uptight first‐time parent… I was really concerned that if he didn't eat five different vegetables in a sitting, then somehow, I had failed as a parent, that I hadn't catered for his nutritional requirements and his life was going to be impacted so adversely… Since then, I've really had to be kind to myself… I go, ‘You know what, my kids eat well, they have a varied diet, they're not fussy eaters.’ Yeah, if they don't like something, I'm far more okay with that than what I was.

### 3.3 Model overview

**Figure 3:**
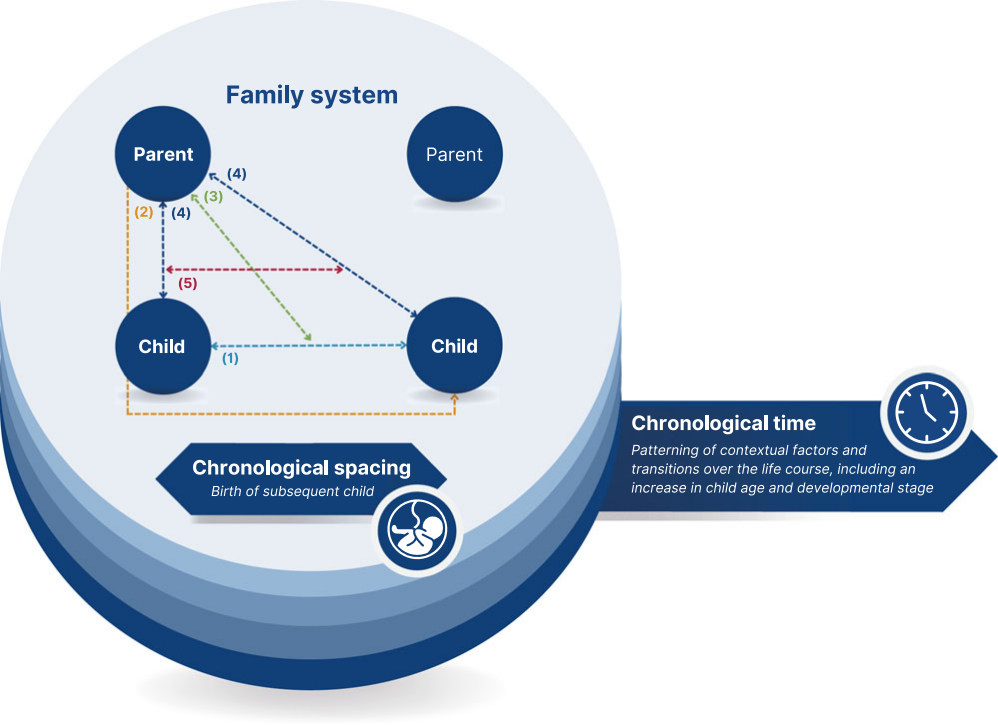
Conceptual model of feeding in the context of siblings. (1) Siblings interacting (2) Parents using siblings as mediators of feeding practices (3) Parents moderating sibling dynamics through feeding practices (4) Parents adapting feeding practices in response to siblings (5) Parents' evolving feeding practices with the inherent restructuring of sibship size and positioning.

A conceptual model was developed to explain how mealtimes are enacted with siblings (Figure 3). This model is comprised of the five interacting processes identified in the data. The extent to which these processes are applied may vary according to the chronological spacing (i.e., age gap) of siblings. For example, parents were often more inclined to role model the behaviour of an earlier‐born child if there was a wider age gap between the siblings. The processes may also evolve over chronological time with changes in contextual factors and transitions over the life course. For example, the transition of a later‐born child into toddlerhood constituted a narrowing gap between siblings' cognitive and physical skills, which often resulted in siblings engaging in more frequent direct interactions, and parents enforcing more consistent expectations between them. While it is acknowledged that each family member contributes to the shared realities of mealtimes, processes were conceptualised only in relation to one parent and two children for the initial development of this model.

## 4 DISCUSSION

Sibling influences are frequently overlooked at mealtimes, despite recognition of their role in child development and adjustment across a broad range of disciplinary perspectives (McHale et al., 2012). Based on empirical mixed‐methods data, this study was the first to broadly conceptualise the enactment of mealtimes in families with siblings. The resulting model describes processes through which siblings may exert *direct* or *indirect* influence over how children are socialised around food during their toddler and preschool age years. Of note is the description of feeding practices used by siblings, such as pressure to eat and overt restriction, which previously had only been described in parents. Furthermore, feeding practices were identified in parents that may occur only in the presence of a sibling, such as vicarious operant conditioning. Thus, aligning with the principles of family systems theory (Broderick, 1993), this study demonstrates the complex, interrelated nature of feeding interactions within families.

### 4.1 Direct sibling influences

Through their frequent and often emotionally intense interactions, siblings may exert *direct* influence over the behaviours of one another (McHale et al., 2012). The current study contributes to existing knowledge by exploring the nature of these interactions within the context of mealtimes. As conceptualised in the broader literature (Howe & Recchia, 2014), sibling interactions in this sample were both reciprocal and hierarchical in nature.

Reciprocal exchanges were observed between siblings, in which they served as mutual companions and competitors. For example, siblings engaged in storytelling and play, which often revolved around food. Through these behaviours, the capacity for siblings to expand one another's social and sensory mealtime experiences was evident. While research exploring the specific impacts of siblings on child eating behaviours is scarce (Ragelienė & Grønhøj, 2020), these interactions may partly explain why children tend to eat *more* in the presence of co‐eaters, particularly when there is a high degree of familiarity among them, such in the case of siblings (Salvy et al., 2008).

The competitive nature of the sibling relationship was also observed and discussed among parents. For example, siblings tended to compete for resources such as food and attention. These dynamics may be interpreted through the lens of social comparison theory (Festinger, 1954), which is premised on the assumption that humans are intrinsically motivated to evaluate themselves based on comparison with others. Due to their close proximity, siblings serve as prime targets of social comparison (Whiteman et al., 2011). Research indicates that children who, for example, receive more criticism or less affection from their parents tend to form ‘upward’ comparisons with their siblings, which may be associated with higher externalising behaviours (Buist et al., 2013). Research also demonstrates that children are not averse to others receiving less than themselves. For example, when tasked with distributing resources, studies have shown that preschool‐aged children tend to prefer advantageous over equal divisions (Sheskin et al., 2014; Smith et al., 2013), which may be due to their decisions being driven mostly by affective rather than cognitive factors (Paulus & Essler, 2020). This tendency may also emerge out of feelings of ‘scarcity’, with previous research indicating that children from larger families may be at higher nutritional risk compared to children from smaller families (Kucera & McIntosh, 1991).

It was evident in the current study that such behaviours may impact the enactment of mealtimes. For example, the formation of food‐based comparisons between siblings occasionally resulted in externalised behaviours such as tantrums and refusals if children perceived that they were at a relative disadvantage to their sibling. On the contrary, children who received less negative attention from their parents during mealtimes, tended to exaggerate preferred eating behaviours, perhaps to ensure their continuation in a more ‘favoured’ position. To minimise competition, however, it is postulated that siblings may also differentiate themselves and adopt identities in line with these comparisons (e.g., as the ‘good’ vs. ‘bad’ eater) (Schachter et al., 1976). This phenomenon was evident in the current study, with some siblings differentiating themselves in terms of their food preferences and eating behaviours.

Hierarchical relationships were also observed, in which the older sibling tended to assume responsibility as a role model for the younger sibling at mealtimes. It was evident in both the mealtime observations and interviews that children tended to imitate sibling behaviours. This finding aligns with principles of social learning, which posits that children have an innate predisposition to observe and imitate the behaviours of others, particularly if there are perceived similarities between them (Bandura, 1986). Parents tended to describe this influence as asymmetrical through reference to their younger child learning to eat via observations of their older sibling. This hypothesis has been supported in the literature. For example, in 4‐ to 8‐year‐old children, the association between last‐born sibling status and higher risk of overweight and obesity was partially mediated by lower food fussiness (Mosli, Lumeng, et al., 2015). This finding indicates that younger siblings may consume more, possibly due to a tendency to imitate the behaviours of older siblings, who may also expose them to a wider variety of foods. Despite this, the younger sibling was often depicted as the ‘fussier’ child by most parents in the current study, indicating that other dynamics may also be at play. For example, it was evident in the current study that the older sibling may be used as a mediator for enacting pressure to eat. With additional pathways for pressure to be exerted, there may be a higher risk of overriding the younger sibling's capacity for self‐regulation, which may contribute to the development of overweight or obesity as this child enters primary school.

Older siblings also tended to assume a parenting role by imitating the feeding practices enacted or endorsed by their parents. Similar dynamics were observed in a sample of mothers and their 4‐ to 8‐year‐old children when a sibling was present during mealtimes (Mosli, Miller, et al., 2015). The authors of that study found that later‐born children tended to receive more encouragement to eat from their sibling when compared to earlier‐born children (Mosli, Miller, et al., 2015). In a subsequent analysis of that sample, encouragement to eat from siblings was positively associated with maternal feeding practices, such as pressure to eat and restriction (Mosli et al., 2016). The current study expands on these findings by demonstrating the broader extent to which children may emulate their parents' feeding practices with siblings. For example, children were observed to enact both responsive (i.e., praise, negotiation, and education) and nonresponsive (i.e., pressure to eat and overt restriction) feeding practices. Research has also demonstrated that a higher degree of concordance between maternal and paternal persuasive and instrumental feeding may be associated with higher food fussiness in children (H. A. Harris et al., 2018). Despite the cross‐sectional design of that research limiting inferences about causation, its finding may be indicative of the accumulative effects of nonresponsive feeding practices when endorsed consistently by both parents. It may therefore be hypothesised that older siblings who imitate these practices may also compound their effects on child behavioural and weight outcomes.

### 4.2 Indirect sibling influences

Siblings may also exert an *indirect* influence on children by virtue of their impact on broader family dynamics and structures, namely via differences in parenting practices (McHale et al., 2012). Feeding practices encompass both provision and socialisation and are used by parents to limit or facilitate food intake in their children (Vaughn et al., 2016). Findings from the current study demonstrate how the enactment of these practices may be altered by the presence of siblings. First, feeding practices incorporating *both* siblings were identified through observation and interview data. These practices occurred over a spectrum of indirect feeding, which ranged from role‐modelling sibling behaviours to leveraging the competitive nature of siblings. For example, some parents attempted to condition their children indirectly by rewarding their sibling for eating, often with food‐related rewards. This practice further draws on principles of social learning, in which it is assumed that children are more likely to adopt behaviours that they have observed if these behaviours have been externally reinforced (Bandura, 1986). The use of instrumental feeding, however, may increase a child's preference for foods used as rewards, such as sweets or desserts (DeCosta et al., 2017). Children with siblings perceived as ‘fussy eaters’ may therefore be exposed to this nonresponsive practice more often, irrespective of their own eating behaviours. Another example of a feeding practice within this construct was the use of comparative food descriptions. This practice describes parents' attempts to foster their child's liking of a food by describing its attributes using comparative and superlative vocabulary (e.g., ‘bigger’ or ‘biggest’). Research has shown that the use of food‐based social comparisons may impact the liking and consumption of food in adults. For example, in an experimental study, adults tended to eat more when told that they were eating a hedonically *better* meal than somebody else (Mills et al., 2020). However, limited research exists on the use of this practice in the context of children. Typically, the practices within this construct were enacted with the intention of modifying the eating behaviours of one child, using their sibling as a mediator. To the authors' knowledge, these practices are yet to be examined within the literature but may provide novel targets for family‐based feeding interventions, and, therefore, warrant further research.

Feeding practices were also shaped by parents' intentions of moderating unfavourable dynamics between the siblings (i.e., by minimising conflict and competition between them). This was most often achieved through parents' attempts to feed siblings similarly. This practice aligns with the social ideal of fairness, which is particularly salient in Western culture, reflective of the current sample (Whiteman et al., 2011). It is, therefore, plausible that research in other cultures may capture differing dynamics. Previous studies have also demonstrated that parents may be motivated to use consistent feeding practices with siblings to mitigate conflict between them (Damen et al., 2020) or avoid upsetting one particular child (Berge, Trofholz, et al., 2016). This ideology may account for similarities in feeding practices observed between siblings in the literature, particularly in relation to food provision (Berge, Trofholz, et al., 2016; Bosk, 1988). However, when responding to food fussiness, parents frequently resort to using nonresponsive indulgent practices, such as catering to food preferences (Fraser et al., 2021). Thus, as implied in the current study, the concurrent use of these practices may have implications for not only the ‘fussier’ child but also their siblings. For example, siblings' exposure to nonpreferred foods (e.g., vegetables) may occur less frequently, irrespective of their own eating behaviours, resulting in reduced dietary variety (Nekitsing et al., 2018). Attempts to enact this ideology may also impact parents' responsiveness to the individual needs of each child despite differences in their appetites, temperaments, or developmental stages.

In the current study, it was evident that the structure of the sibling dyad may play into these dynamics. For example, parents of children with a wider age gap appeared to have age and developmentally appropriate expectations for each of their children. As such, parental differential treatment may be more pronounced for nontwins compared to twins, with variations mostly accounted for by the age gap between the siblings (Mönkediek et al., 2020). The current study also identified competing priorities, such as concerns over a child's weight, which may generate apprehension among parents. This finding aligns with other literature demonstrating that concerns about child growth may result in parents adapting their use of certain feeding practices, such as restriction (Berge, Trofholz, et al., 2016; Bosk, 1988; Payne et al., 2011). Navigating competing priorities within the context of siblings may therefore serve as a relevant target of feeding interventions.

Furthermore, differences in parent feeding practices identified between siblings were often attributed to differences in their eating behaviours. For example, some parents pressured the fussier sibling to eat more. In parallel to this, some parents imposed more restrictions on the sibling who exhibited a higher tendency to overeat. These findings correspond with other literature, indicating that parents' use of more controlling feeding practices, including pressure to eat, overt restriction, and food rewards, may be contingent upon the weight status, eating behaviours, temperaments, or birth order of siblings (Ayre et al., 2022). In the current study, however, parents were less inclined to differentiate their children based on weight status. This may be due to the relative ages of children included within the current sample, with parental discrimination of child weight status tending to be less accurate in toddler and preschool‐aged children (Huang et al., 2007). Moreover, in the current study, differential feeding was evident not only in direct parent–sibling interactions but also in the broader mealtime structure, which may have implications for the practicalities of executing family mealtimes. For example, some parents served siblings at varying times depending on their appetites or offered different foods to siblings to cater for their preferences. While it has been recognised that parents tend to compare their children against one another in the context of feeding (Webber et al., 2010), findings from the current study also demonstrate how these comparisons may impact the value judgement that parents place on their children. As differential treatment has been associated with adverse effects on child behavioural adjustment in the broader parenting literature (Oliver & Pike, 2018), further research on the effects of differential feeding in relation to both responsive and nonresponsive practices is needed.

In the current study, parents further described how their feeding practices had evolved with the inherent restructuring of sibship size and positioning that occurred following the birth of their younger child. The findings corroborate recent work by Ruggiero et al. (2021) relating to processes of resource dilution and learnt experience, and their role in parent feeding. First, parents acknowledged that finite resources such as time and attention had to be divided among siblings. In accordance with this finding, it has been inferred within the literature that the process of resource dilution may account for inverse associations between the number of children and perceived feeding responsibility among mothers (Mosli, 2021). It was evident in the current study, however, that some resources may be reallocated or renegotiated, rather than simply diluted. For example, tasks involved in executing the mealtime, such as setting and clearing the table, were often delegated among children. As discussed earlier, siblings also facilitated mealtime interactions, which may have further minimised demands on parents. However, for more resource‐intensive tasks such as grocery shopping and meal preparation, the presence of multiple children often served as a barrier to their involvement. This finding may relate to perceptions of increased household chaos following the birth of a later‐born child, particularly if there is a lack of social support received while undertaking these tasks due to competing parental work schedules (Ruggiero et al., 2023). As the involvement of children in these tasks is associated with healthier dietary intakes (Metcalfe & Fiese, 2018), it is pertinent that feeding interventions are designed with strategies aimed at mitigating the impacts of resource dilution following the birth of subsequent siblings.

Second, parents acknowledged shifts in their attitudes towards feeding, which were reminiscent of the notion of learnt experience as described by Ruggiero et al. (2021). For example, with the novelty of the firstborn child, parents were reportedly more anxious about feeding. However, it was through this experience that parents found reassurance in their child's capacity for appetite self‐regulation, which enabled them to engage in more responsive feeding practices with their later‐born child, despite resource dilution. Similarly, mothers of toddlers have reported increases in their knowledge and self‐efficacy with feeding over time (Damen et al., 2020; Ruggiero, Moore, et al., 2022), which may also account for higher levels of responsive feeding observed in later‐born, compared to first‐born children (Ruggiero, Marini, et al., 2022).

### 4.3 Implications for research and practice

This study provides a conceptual model that maps sibling‐related processes integral to the enactment of mealtimes within families. Siblings are frequently overlooked in explanations of the effects of family dynamics on child behavioural and health outcomes. Therefore, this model expands knowledge of the operation of families as socialising systems at mealtimes and lays the foundation for future intervention research. This is pertinent considering that periods of transition, such as the birth of subsequent siblings, may provide effective opportunities for intervention (Cicchetti & Toth, 1992). Targeting siblings may also provide a cost‐effective and less stigmatising gateway into feeding dynamics than focusing on parent‐child dyads. Opportunities may include the use of anticipatory guidance aimed at reinforcing principles of responsive feeding, particularly for parents whose perceptions or expectations of their children differ. For example, interventions may incorporate advice relating to feeding practices only applicable to parents of siblings, such as leveraging sibling competitiveness to persuade a child to eat. Reinforcing these principles may also mitigate or capitalise on the potentially compounded effects of feeding practices that are imitated by siblings. In addition, interventions may include tailored advice for parents to effectively adapt to later‐born children who exhibit different characteristics. This may include, for example, the integration of strategies for occupying a child who eats more quickly without disrupting the mealtime of their sibling. However, these opportunities necessitate further research into feeding within the context of siblings, such as measuring and evaluating practices in which siblings are used as mediators or exploring the implications of differential feeding on child behaviours.

### 4.4 Strengths and limitations

This study employed principles of CGT to examine mixed‐methods data on the enactment of mealtimes in families with multiple children. Using naturalistic mealtime observations as an adjunct to semistructured interviews served to enhance the depth and breadth of knowledge constructed by offsetting the limitations inherent in each method. For example, while the interviews captured the attitudes and intentions of parents towards feeding, the observations enabled the exploration of social interactions between parents *and* children, which contributed to the shared realities of mealtimes in families. Furthermore, by using GCT methods, the authors were informed, but not constrained by existing conceptualisations of feeding in the literature. This enabled the exploration of nuanced interactions, which contribute novel findings to the field.

However, the study was not without limitations. First, despite attempts to direct recruitment towards a diverse socioeconomic population, the sample was confined mostly to white, educated, two‐parent families. While sampling in CGT is driven by theory construction as opposed to population representativeness (Charmaz, 2014), the recruitment of a demographically diverse sample may have captured nuances in how the model may be applied across different family cultures and structures. Future research is therefore needed to examine, for example, how processes such as resource dilution may affect single‐parent or food‐insecure families, or the degree to which parents may differentially preference male siblings in cultures deeply rooted in patriarchal systems (Le & Nguyen, 2022). Finally, due to feasibility constraints, repeat interviews and member checking were not undertaken. However, methods to increase the validity of the findings included prolonged engagement with participants, multiple methods of data collection, and extensive use of participant quotations (Morse, 2015).

## 5 CONCLUSION

The model constructed in this study provides a broader conceptual understanding of the enactment of mealtimes in families with siblings. The five interacting constructs in this model describe both direct and indirect processes through which siblings may impact the socialisation of children around food during their toddler and preschool age years. Of note is the complex and integral role of siblings in determining how parents navigate feeding, which provides opportunities for future exploratory and intervention research.

## AUTHOR CONTRIBUTIONS

All authors contributed to the conceptualisation and design of the study. Susannah K. Ayre completed data collection and analysis, with input received from all authors. Susannah K. Ayre drafted the manuscript, and all authors reviewed and approved the final version.

## CONFLICT OF INTEREST STATEMENT

The authors declare no conflict of interest.

## Supporting information

Supporting information.

## Data Availability

The data that support the findings of this study are available from the corresponding author upon reasonable request.
