# Supplementary material for: ‘I'm having jelly because you've been bad!’: A grounded theory study of mealtimes with siblings in Australian families
Source: Matern Child Nutr. 2023 Feb 19;19(2):e13484. doi: 10.1111/mcn.13484 (PMC10019066; doi:10.1111/mcn.13484)
Supplement: Supplementary file 1 — Supporting information. [file MCN-19-e13484-s001.docx]

***Supplementary Material***

[Table S1: Consolidated Criteria for Reporting Qualitative Research (COREQ) Checklist 2](#_Toc117753486)

[Table S2: Semi-structured interview protocol 4](#_Toc117753487)

[Table S3: Evidentiary quotations of parents adapting their feeding practices based on differences in sibling characteristics 6](#_Toc117753488)

# *Table S1:* Consolidated Criteria for Reporting Qualitative Research (COREQ) Checklist

| **Topic** | **Item** | **Guide Questions/Description** | **Page** |
| --- | --- | --- | --- |
| **Domain 1: Research team and reflexivity** | | | |
| *Personal characteristics* | | | |
| Interviewer/facilitator | 1 | Which author/s conducted the interview or focus group? | 5 |
| Credentials | 2 | What were the researcher’s credentials? (e.g., PhD, MD) | 5, 9 |
| Occupation | 3 | What was their occupation at the time of the study? | 5 |
| Gender | 4 | Was the researcher male or female? | 5 |
| Experience and training | 5 | What experience or training did the researcher have? | 5-6 |
| *Relationship with participants* | | | |
| Relationship established | 6 | Was a relationship established prior to study commencement? | 6 |
| Participant knowledge of the interviewer | 7 | What did the participants know about the researcher? (e.g., personal goals, reasons for doing the research) | 6 |
| Interviewer characteristics | 8 | What characteristics were reported about the interviewer/facilitator? (e.g., biases, assumptions, interests in the research topic) | 6 |
| **Domain 2: Study design** | | | |
| *Theoretical framework* | | | |
| Methodological orientation and theory | 9 | What methodological orientation was stated to underpin the study? (e.g., grounded theory, discourse analysis, ethnography, phenomenology, content analysis) | 5 |
| *Participant selection* | | | |
| Sampling | 10 | How were participants selected? (e.g., purposive, convenience, consecutive, snowball) | 7 |
| Method of approach | 11 | How were participants approached? (e.g., face-to-face, telephone, mail, email) | 7 |
| Sample size | 12 | How many participants were in the study? | 6 |
| Non-participation | 13 | How many people refused to participate or dropped out? What were the reasons? | 11 |
| *Setting* | | | |
| Setting of data collection | 14 | Where was the data collected? (e.g., home, clinic, workplace) | 8-9 |
| Presence of non-participants | 15 | Was anyone else present besides the participants and researchers? | 6  Table 2 |
| Description of sample | 16 | What are the important characteristics of the sample? (e.g., demographic data, dates) | 11 Table 1 |
| *Data collection* | | | |
| Interview guide | 17 | Were questions, prompts, guides provided by the authors? Was it pilot tested? | Supplementary Table 2  NA |
| Repeat interviews | 18 | Were repeat interviews carried out? If yes, how many? | NA |
| Audio/visual recording | 19 | Did the research use audio or visual recording to collect the data? | 8-9 |
| Field notes | 20 | Were field notes made during and/or after the interview or focus group? | 8 |
| Duration | 21 | What was the duration of the interviews or focus groups? | Table 2 |
| Data saturation | 22 | Was data saturation discussed? | 8 |
| Transcripts returned | 23 | Were transcripts returned to participants for comment and/or correction? | NA |
| **Domain 3: Analysis and findings** | | | |
| *Data analysis* | | | |
| Number of data coders | 24 | How many data coders coded the data? | 10-11 |
| Description of the coding tree | 25 | Did authors provide a description of the coding tree? | NA |
| Derivation of themes | 26 | Were themes identified in advance or derived from the data? | 10 |
| Software | 27 | What software, if applicable, was used to manage the data? | 9-10 |
| Participant checking | 28 | Did participants provide feedback on the findings? | NA |
| *Reporting* | | | |
| Quotations presented | 29 | Were participant quotations presented to illustrate the themes/findings? Was each quotation identified? (e.g., participant number) | 12-30 |
| Data and findings consistent | 30 | Was there consistency between the data presented and the findings? | 12-30 |
| Clarity of major themes | 31 | Were major themes clearly presented in the findings? | 30-31 Figure 3 |
| Clarity of minor themes | 32 | Is there a description of diverse cases or discussion of minor themes? | 12-30 |

# *Table S2:* Semi-structured interview protocol

| Number | Questions | Follow up or alternative questions | Prompts |
| --- | --- | --- | --- |
| 1 | How would you describe [Sibling 1] and [Sibling 2] in terms of their characteristics? | How are they similar and different from one another? | Eating behaviours, appetites, food preferences, temperaments, personalities, developmental abilities, body size/weight |
| 2 | What do mealtimes in your home look like? | Some mealtimes can feel more difficult than others. How would you describe a more difficult mealtime? | Setting, timing, atmosphere, presence of family members, rules, rituals |
| 3 | What do you think the role of a parent should be at mealtimes? | What are your own priorities at mealtimes? |  |
| 4 | How do you approach feeding with [Sibling 1] and [Sibling 2]? | How do you decide on what to feed them? What would you do in a situation where they are refusing to eat? What would you do in a situation where they are eating too much food (e.g., sweets)? What is similar and different in your approach for each of them? |  |
| 5 | How has your experience of mealtimes changed since having [Sibling 2]? † | What did you learn from your experience with feeding [Sibling 1]? What has been easier or more difficult since having [Sibling 2]? **Alternative question:** How did the prospect of having twins impact your expectations of feeding at mealtimes? † |  |
| 6 | When it comes to influencing what or how much either one of your children eat, what role do you think their sibling plays? † | Are there any strategies that you use at mealtimes that can only be used when both siblings are present? |  |
| 7 | How do you go about dividing resources between [Sibling 1] and [Sibling 2] at mealtimes? † | How has this impacted how each of them experience feeding? | Time, attention, food, utensils |
| 8 | How do you mitigate or manage conflict between [Sibling 1] and [Sibling 2] at mealtimes? † |  |  |
| 9 | Is there anything you would like to clarify or add? |  |  |

† Questions included as the result of theoretical sampling

# *Table S3:* Evidentiary quotations of parents adapting their feeding practices based on differences in sibling characteristics

| Sibling characteristic | Interview excerpt | |
| --- | --- | --- |
| Developmental stage | Family 7 (4-year-old boy and 2-year-old boy): | |
|  | *Mother:* | *“Age, I take into consideration a little bit and go, ‘Well, you're two and you're still exploring food, that's okay.’ But yeah, I'm starting to really go, ‘Okay, well, no, broccoli is what you have to eat…’ It's a non-negotiable for Oliver… [For] Liam, up until fairly recently, I was a little bit more relaxed about it.”* |
| Eating behaviours | Family 3 (5-year-old girl and 2-year-old boy): | |
|  | *Mother:* | *“With Miles, because he’s fussy… [we’ll] just offer him choices, as opposed to Harper, we’ll put it on the plate, and she’ll eat it… Generally, I tell Harper, ‘This is it.’ …Whereas with Miles, it’s literally just giving him a range of options of the stuff we know he eats.”* |
|  | Family 10 (5-year-old boy and 2-year-old boy): | |
|  | *Mother:* | *“I know, if I put strawberries on Henry’s dinner plate, he’ll just eat the fruit and will ignore anything else. Whereas George will leave that for his dessert. So George might have a strawberry on his, but Henry won’t until after, and that sort of thing… It’s just trying to put a plate in front of them that controls behaviour.”* |
|  | Family 14 (5-year-old girl and 3-year-old girl): | |
|  | *Mother:* | *“Isabel, from a food perspective, we say she knows her tummy. She never overeats… Mia, the three-year-old, will eat more and she needs to be rationed a little bit more… Something with Mia we're very conscious of [is] if we go out and eat, we take away the chips, halve the chips, or even ask for no chips… 'cause she'll just eat them all… With Mia, I'll say things like, ‘Have one chippy’ …or ‘What is your tummy say? I think you've had enough of this.’”* |
| Temperaments | Family 15 (5-year-old boy and 3-year-old girl): | |
|  | *Mother:* | *“With Evie, I guess she’s usually pretty happy to sit and eat... With Max, [it’s] very hard to keep him at the table. Yeah, he’ll obviously tantrum and stuff if he’s forced to sit at the table and it’s really not worth it for us. So we just let him go a little bit but try and encourage him back to the table.”* |
| Food preferences | Family 10 (5-year-old boy and 2-year-old boy): | |
|  | *Mother:* | *“I know Henry won’t eat tomatoes, so I might give him one, and give George three. But I know Henry will eat plenty of cucumbers. So I’ll give him more than I give George.”* |
| Contextual factors | Family 18 (5-year-old boy and 3-year-old girl): | |
|  | *Mother:* | *“We know where their limits are, and we work at pushing them just a little bit further in their limits to try new things. [It's] based on how their day has gone… For example, Layla might have had a really rough day, or she might have bumped her head while out riding her bike, or something like that. We know that she's at her capacity already, so we won't push her much. So we look at all the factors that have gone into what has led up to dinner time as well… I guess that's probably the biggest difference in approach with each kid.”* |
